# Weak associations between personality and contraceptive choice

**DOI:** 10.3389/fnins.2022.898487

**Published:** 2022-10-27

**Authors:** Belinda Pletzer, Carmen Lang, Birgit Derntl, Ramune Griksiene

**Affiliations:** ^1^Department of Psychology and Centre for Cognitive Neuroscience, University of Salzburg, Salzburg, Austria; ^2^Department of Psychiatry and Psychotherapy, Tübingen Center for Mental Health, University of Tübingen, Tübingen, Germany; ^3^Department of Neurobiology and Biophysics, Life Sciences Center, Vilnius University, Vilnius, Lithuania

**Keywords:** hormonal contraceptives, personality, gender role, masculinity, femininity, adverse mood effects

## Abstract

Prospective randomized controlled trials on hormonal contraceptive (HC) effects on the brain are rare due to a number of methodological challenges. Thus, much of the evidence on HC effects on the brain comes from cross-sectional studies comparing HC-users to non-users. In interpreting these findings, it is of importance to be aware of potential confounds associated with women’s contraceptive choices. Previous studies have discussed age, education, social status, sexual orientation, relationship status, and tolerability of HC. Given the current trend toward a reduction in HC use and increased skepticism toward HC it seems relevant to also identify variables associated with women’s attitudes toward HC and whether they may represent confounds for neuroscientific studies. In the present study, we investigated whether women’s personality characteristics were associated with their choice to use or not use HC in the present, past and future and the type of HC chosen. 1,391 females aged 18–45 years participated in an online survey including the HEXACO-60 personality questionnaire, as well as two different measures of gender role, and provided information about their current and previous contraceptive status, as well as experiences with and attitudes toward contraceptive use. We compared (i) current, previous and never-users of HC, (ii) prospective users of HC to women who opposed future HC use, and (iii) current users of IUDs to current users of oral contraceptives. Results revealed that associations between personality and the decision to use or not use HC were negligible, while differences in personality were observed corresponding to contraceptive type. Current users of IUDs showed higher agreeableness and extraversion compared to current users of oral contraceptives. The results suggest that personality is more strongly associated to the choice of contraceptive type rather than the choice between hormonal and non-hormonal options.

## Introduction

Hormonal contraceptives (HC), and in particular combined oral contraceptives (COC), have been linked to changes in brain structure and function (Porcu et al., 2019; Brønnick et al., 2020; Rehbein et al., 2021) and associated with behavioral changes, including women’s mental health (Sundström-Poromaa, 2021), cognitive performance (Warren et al., 2014), mate preferences (Alvergne and Lummaa, 2010), and social and emotional functioning (Montoya and Bos, 2017; Lewis et al., 2019). Considering the wide-spread use of HC, societal consequences have been intensively discussed (Alvergne and Lummaa, 2010; Montoya and Bos, 2017; Sundström-Poromaa, 2021). Given the estrogenic actions of ethinylestradiol and other synthetic estrogens (Stanczyk et al., 2013), as well as the progestogenic, androgenic, anti-androgenic, or mineralocorticoid properties of the various synthetic progestins contained in HC (Sitruk-Ware, 2006; Griksiene et al., 2022), the described effects appear plausible. Steroid actions on the brain from neurogenesis over synaptic transmission (Barth et al., 2015) to the modulation of large-scale brain networks are well documented (Hidalgo-Lopez et al., 2021). With the increasing availability and usage of long-lasting methods like hormonal intra-uterine devices (IUD), more studies are also conducted comparing the effects of various methods of HC, e.g., COC vs. IUD on the brain and behavior (Bürger et al., 2021).

However, due to the variety of methodologies employed in studies of HC actions on brain and behavior, it is hard to disentangle the effects of various combinations of synthetic steroids from confounding factors. Most importantly, the majority of HC studies uses a cross-sectional design, comparing COC-users to non-users, different groups of COC-users, or COC-users and IUD-users. These groups may differ in a range of factors correlated with the contraceptive choice, e.g., age, education, socio-economic status, relationship status, or tolerability of HCs (Pletzer and Kerschbaum, 2014). The selection of birth control method might depend on women’s socioeconomic, demographic, or partnership characteristics (Eeckhaut et al., 2014) as well as on a woman’s personal preferences (Dragoman, 2014). While demographic differences between the groups can usually be well-controlled, tolerability of HC and personality traits are generally not documented. However, those two factors in particular are highly relevant to the dependent variables studied in contemporary HC research, i.e., brain structure and function, as well as mood, cognition, and wellbeing.

Regarding the tolerability of HC, the so-called survivor-effect may introduce a sampling bias in cross-sectional study designs. While long-term users usually tolerate HC well, non-users have usually stopped using HC due to adverse side effects (Oinonen and Mazmanian, 2002). Most commonly, emotional side effects and weight gain are listed as a reason for discontinuation of HC-treatment (Lindh et al., 2009). The neurophysiological factors that determine the tolerability of HC are currently unknown. Accordingly, there may be pre-existing neurophysiological differences between HC-users and non-users, such that the differences found in cross-sectional designs may not actually be a result of HC-use, but rather affect the choice to use HC or not. Accordingly, the contraceptive history of non-users, their reasons for discontinuation, as well as their side effect profiles are relevant factors to consider in cross-sectional studies on HC-use.

Relatedly, HC-use has dropped in the past years, which may on the one hand be related to the availability of non-hormonal options, like copper IUDs. However, concerns about potential long-term effects on (mental) health and fertility have resulted in increasing skepticism among women regarding synthetic steroids (e.g., Fiala and Parzer, 2019; Landersoe et al., 2019; Svahn et al., 2021). Thus, newer studies may well face an additional bias concerning women’s attitudes toward HC-use. Accordingly, pre-existing differences between HC users and non-users may not only concern neurophysiological factors determining contraceptive tolerance, but also psychological factors, including personality traits related to women’s choice to use HC.

Personality traits have been associated with differences in performance on cognitive tasks (Aschwanden et al., 2020), socioemotional functioning (Canli et al., 2002; Yang et al., 2021), psychopathologies (Kotov et al., 2010) and brain structure (Nostro et al., 2017). For example, open, extraverted, and emotionally stable participants demonstrated better verbal fluency (Sutin et al., 2011); neuroticism (negatively) and openness (positively) affected self-estimates of spatial and logical abilities (Stieger et al., 2010); higher conscientiousness, openness, and extraversion as well as lower neuroticism were associated with better memory performance (Luchetti et al., 2021). With regard to socioemotional functions, high neuroticism scores were related to decreased brain activation in the medial prefrontal cortex during implicit negative emotion processing (Yang et al., 2021), while a higher degree of extraversion correlated positively with amygdala activation to happy facial expressions (Canli et al., 2002). And in terms of “big” personality traits (i.e., BIG-5 neuroticism, extraversion, openness, agreeableness, and conscientiousness), especially neuroticism (high) and conscientiousness (low) were significantly associated with anxiety, depressive, and substance use disorders (Kotov et al., 2010). In addition, women’s personality traits may be related to a way of coping with physical and/or psychological discomfort determined by hormonal fluctuations during the menstrual cycle. Therefore, women who are more vulnerable may be choosing HC to avoid menstrual cycle related inconvenience. For example, it was demonstrated that women with high neuroticism score were more likely to use hormone replacement therapy (as a way of coping with menopause symptoms) (Loekkegaard et al., 2002).

To the best of our knowledge, only few studies have examined associations between HC-use and women’s personalities, yielding inconsistent results. An early study by Beard et al. (1974) demonstrated a negative relationship between neuroticism scores and the reliability of contraceptive methods used by study participants. It was demonstrated that women with the lowest neuroticism scores tended to use the most reliable methods of contraception (pills and IUDs), whereas participants scoring highest on neuroticism did not use any form of contraception. Priestnall et al. (1978) reported that OC-users were significantly less positive toward religion, more linked to feminism and less neurotic than non-users. No differences were found between users and non-users with regard to extraversion in that study. Jacobsson et al. (1981) demonstrated that the long-term COC- or IUD-users were more stable psychologically and exhibited a lower neurotic potential. However, a more recent study by Ross et al. (2001) demonstrated the opposite result, i.e., significantly higher neuroticism in COC-users than in non-users. Finally, the most recent studies (Hamstra et al., 2017; Beltz et al., 2019) did not find significant differences in personality scores between OC users and non-users.

There are multiple potential reasons for these inconsistencies. On the one hand, women’s attitudes toward HC and the demographic and socio-economic characteristics of HC-users have changed over time and are also subject to cultural differences. Similarly, the composition of the comparison group of naturally cycling women may have contributed to inconsistencies in the results, especially if previous experiences with HC were not controlled for. Accordingly, a clearer differentiation between never users, previous users and prospective users of HC among the naturally cycling group will aid to adequately capture associations between personality and women’s attitudes toward HC.

On the other hand, it has been discussed, especially with respect to gender differences, that broad personality factors, like the BIG-5, may not be adequately sensitive to group differences (Del Giudice et al., 2012). They encompass a variety of facets, which may be differentially related to the grouping variable, thereby averaging out the group differences in the overarching factor. It is possible, that a similar situation occurs with respect to HC-use or HC type. Thus, the use of a more fine-grained instrument, allowing the simultaneous assessment of broad personality factors and their underlying facets may provide a clearer picture.

Particularly, the sub-facets of extraversion, like dominance and warmth, show gender differences in opposite directions (Del Giudice et al., 2012), given their association with the gender roles masculinity and femininity, respectively (Eagly and Sczesny, 2019). Indeed, various gender-sensitive facets of extraversion, agreeableness and neuroticism may not only be grouped according to the BIG-5, but also along the overarching dimensions of masculinity and femininity (Gruber et al., 2019). Given that femininity in particular has been associated with gray matter volumes in prefrontal areas (Pletzer, 2019), while masculinity has repeatedly been related to spatial abilities (Reilly and Neumann, 2013; Beltz et al., 2022), it is an interesting question whether HC choice is also associated with femininity or masculinity. So far, two studies have investigated associations between HC-use and women’s gender role self-concept with inconsistent results: While one study demonstrates that HC-users rate themselves as more feminine compared to non-users (Pletzer et al., 2015), the other study demonstrates no differences in the gender role self-concept of HC-users and non-users (Nielson and Beltz, 2021). The two studies differ in the gender role measures employed. While Pletzer et al. (2015) used only subjective rating scales, Nielson and Beltz (2021) also asked for instrumental and expressive traits associated with masculinity and femininity. Given that gender roles are a concept with considerable cultural differences (Eagly and Sczesny, 2019), self-concepts may be colored by participants perceptions of what is masculine or feminine. Accordingly, a combination of measures is advisable when assessing gender-role in a cross-cultural context.

To obtain a varied picture of personality traits in current, past and never-users of HC, as well as between COC-users and IUD-users, we chose the HEXACO-60 (Ashton and Lee, 2009), which assesses the BIG-5 personality factors, but allows for differentiation of sub-facets, and further used two different gender role measures. The first question of the present study was whether women’s attitudes toward the use of synthetic hormones for contraception in general are associated with personality factors. To address this question, we compared current, past and never-users of HC, as well as prospective HC-users to women for whom future HC-use is not an option. Based on previous work we hypothesized that neuroticism and femininity may differ between those groups, though the directionality is unclear given inconsistent results. The second question was, whether the type of HC chosen is related to women’s personality. Given that the regular daily intake of COCs requires a greater amount of organization than long-acting methods like IUDs, we hypothesized that conscientiousness is higher in women who choose COCs compared to women who choose IUDs.

## Materials and methods

### Participants

1,391 biologically female women aged 18–45 (mean age: 26 years, SD = 8 years) participated in this study and filled out an online questionnaire on their current and previous contraceptive status, as well as experiences with and attitudes toward contraceptives. Demographic information of participants can be found in Table 1. To determine sex and gender, participants were independently asked, which sex they were assigned at birth and whether they identified as man or woman. Participants were recruited via social media and university emails at the Universities of Salzburg, Tübingen, and Vilnius. Due to the anonymous nature of the study, participants were not compensated for their participation. The study was approved by the University of Salzburgs’ ethical committee.

**TABLE 1:** Comparison of demographic variables between current and previous HC-users and never users prior to matching.

		Never-users (*n* = 321)	Previous users (*n* = 493)	Current users (*n* = 577)	Comparison
Language*^a^*	Lithuanian	97 (30%)	94 (19%)	94 (16%)	***p*** ** < 0.001**
	German	224 (70%)	399 (81%)	483 (84%)	
Age*^b^*		24.85 ± 5.82	27.44 ± 6.64	24.20 ± 5.89	***p*** ** < 0.001**
Handedness*^a^*	Left-handed	35 (11%)	41 (8%)	48 (8%)	*p* = 0.362
Education*^c^*	Apprenticeship	3 (1%)	10 (2%)	4 (1%)	***p*** ** < 0.001**
	Middle school	4 (1%)	5 (1%)	14 (2%)	
	High school	143 (46%)	160 (33%)	299 (52%)	
	University	158 (51%)	316 (64%)	247 (43%)	
	Unknown	13 (4%)	2 (0%)	2 (0%)	
Employment status*^c^*	Employed full time	67 (21%)	142 (29%)	127 (22%)	***p*** ** < 0.001**
	Employed part time	51 (16%)	108 (22%)	104 (18%)	
	In education + part time	31 (10%)	70 (14%)	73 (13%)	
	In education + unemploy.	62 (19%)	53 (11%)	87 (15%)	
	Unemployed	110 (34%)	120 (24%)	186 (32%)	
Sexual orientation*^b^*	Homosexual	18 (6%)	17 (3%)	18 (3%)	***p*** ** = 0.006**
	Bisexual	72 (22%)	117 (24%)	109 (19%)	
	Heterosexual	231 (72%)	359 (73%)	450 (78%)	
Relationship	In a relationship*^a^*	179 (56%)	367 (74%)	444 (77%)	***p*** ** < 0.001**
	Duration*^b^*	5.06 ± 4.72	5.77 ± 5.65	4.13 ± 4.72	***p*** ** < 0.001**
	Satisfaction*^b^*	8.60 ± 1.65	8.55 ± 1.51	8.72 ± 1.49	*p* = 0.320
Children*^a^*		42 (13%)	91 (19%)	47 (8%)	***p*** ** < 0.001**

*^a^*binary variables, compared via *X*^2^-Tests, *^b^*continuous variable, compared via one-way-ANOVAs, *^c^*ranked variables, compared via Kruskall-Wallis tests. *p*-values were FDR-corrected for multiple comparisons. Bold *p*-values indicate significant difference.

### Questionnaires

Questionnaires were presented as part of an online survey via LimeSurvey and presented in German or Lithuanian translations. Data were collected between July 20^th^ to November 15^th^ 2021, i.e., during a time when no COVID19-lockdowns were in place in any of the participating countries.

#### Contraception questionnaire

Participants started the Online Survey by answering several questions about their current and previous contraceptive use. Women who used HC at the time of testing gave information on the duration of their use, side effects, why they chose this form of contraception and whether the start of their use was connected to the beginning of a new relationship.

Naturally cycling women were asked for the reason they decided against using HC. If their response was that there was no need for contraception in general, they were asked whether they could or could not imagine using HC in the future and why. They were also asked to give information on previous HC-use.

#### HEXACO-60

Personality traits were investigated by the self-report form of the HEXACO-60 (Ashton and Lee, 2009) in its German and Lithuanian translations. The inventory consists of 60 items corresponding to the six dimensions of the HEXACO model of personality structure (Honesty-Humility, Emotionality, Extraversion, Agreeableness, Conscientiousness, and Openness to Experience). Participants rated their agreement to each of the statements about themselves (e.g., “I would be quite bored by a visit to an art gallery.”) on a 5-point Likert scale, ranging from 1 (= strongly disagree) to 5 (= strongly agree). The HEXACO also allows a more fine-grained assessment of personality by providing scores of four sub-factors for each of the main personality domains. For the German version of the HEXACO-60, Moshagen et al. (2014) confirmed the 6-factor solution, as well as measurement invariance with respect to gender and report good internal consistencies ranging from 0.74 to 0.83, as well as retest reliability over 7 months of 0.72–0.90. Furthermore, the instrument is well-validated with low correlations between subscales, high correlations to other personality questionnaires (Moshagen et al., 2014), as well as lexical personality factors (Ashton et al., 2007). For the Lithuanian version of the HEXACO-60, the 6-factor solution, as well as construct validity was confirmed by Truskauskaitė-Kunevičienė et al. (2012), who also report good internal consistencies ranging from 0.66 to 0.80. Measurement invariance of the HEXACO-60 with respect to the language/country was recently confirmed in a large-scale confirmatory factor analysis by García et al. (2022).

#### Gender related attributes scale

The Gender Related Attributes Scale (GERAS) developed by Gruber et al. (2019) was used to assess gender role. This scale measures characteristics that are generally perceived as typically masculine or feminine on the three subscales personality, cognition, and interests. All items are rated on a 7-point Likert scale.

First, participants were asked to compare themselves to the general population in how often they portrayed 10 stereotypically masculine (e.g., brave, dominant) and 10 stereotypically feminine (e.g., compassionate, anxious) personality traits on a scale from 1 (= never) to 7 (= always). On the second subscale, participants rated how easy they would find completing each of 14 cognitive tasks on a scale of 1 (= not at all) to 7 (= very). Seven of these tasks required skills that according to previous research men show stronger performance in (e.g., finding an address), whereas the other seven items are typically easier for women (e.g., remembering names and faces).

Finally, participants stated how much they enjoy each of 16 activities on a scale from 1 (= not at all) to 7 (= very much). Again, eight of these items described activities typically perceived as masculine (e.g., watching sports) and eight items described activities typically perceived as feminine (e.g., Yoga). Averaged scores for masculine and feminine items were computed for each subscale. Overall masculinity and femininity scores were obtained by averaging the masculinity and femininity scores of each subscale.

The factorial structure of global masculinity and femininity scores with subscores in personality, cognition and interests, as well as measurement invariance with respect to gender was confirmed by Gruber et al. (2019) for the German version. Gruber et al. (2019) also reports good reliability with Revelle’s Omega, split-half and retest reliability ranging from 0.80 to 0.88. The GERAS was validated against other gender role questionnaires, self- and peer-reports, as well as chosen occupation (Gruber et al., 2019). Translation to Lithuanian was performed by 10 independent German/Lithuanian bilingual native speakers and validated by back-translation. Psychometric properties of the Lithuanian translation have not yet been published.

#### Six-item-scale

To additionally obtain a subjective measure of masculinity and femininity, gender role was also assessed by a Six Item Scale (Pletzer et al., 2015). Participants directly indicated how masculine or feminine they perceived themselves compared to men, other women, and the general population on a scale of 1 (= not at all) to 9 (= very). By measuring subjective masculinity and femininity, this scale takes into account possible cultural and personal differences in what the participant views as typically masculine or feminine.

### Statistical analysis

Data were analyzed using IBM SPSS Statistics 27. Given the large sample size, normality of distributions was determined graphically, using stem- and-leaf plots, histograms, as well as Q-Q plots. All HEXACO-60 and GERAS scales were normally distributed and thus suitable for parametric analysis. The significance threshold was set to p_*FDR*_ < 0.05 throughout the manuscript.

Taking into consideration the whole sample, significant group differences between current, previous and prospective HC-users as well as never-users were observed in language/country, age, education, employment status, sexual orientation, relationship status, relationship duration, and number of children (compare Table 1), resulting in a large number of confounding variables when comparing these groups with respect to personality and gender role. Due to the significant group differences in demographic variables, ANCOVA requirements are violated (Miller and Chapman, 2001; Verona and Miller, 2015). Accordingly, we opted for *a priori* matching of confounding variables between groups using propensity scores. Nevertheless, ANCOVA results are reported in Supplementary Table 3. Propensity score matching is particularly useful, when multiple confounding variables need to be considered (see e.g., Benedetto et al., 2018). Given that never-users were the smallest group (*n* = 321), we assessed current and previous HC-users according to their similarity to never-users. To that end, we performed two binary logistic regression analyses with group (never vs. previous; never vs. current) as dependent variable and age, language, sexual orientation, and relationship status as regressors. Education and socio-economic status were collinear to age and additional inclusion of education in the binary logistic regression did not improve matching. Likewise, relationship duration and number of children were collinear to relationship status. Probabilities of belonging to the never-user group based on those variables (propensity scores) were saved and the 321 current and 321 previous users with the highest probabilities were selected for further analysis.

For comparison of potential future HC-users (*n* = 73) and women who said that future HC-use was not an option for them, 73 of 714 women for whom future HC-use was not an option were selected based on propensity scores for belonging to the future HC group based on age, language, sexual orientation, and relationship status. Finally, for comparison of different HC types, 94 out of 428 COC users were selected as comparison group for 94 IUD users based on propensity scores for belonging to the IUD group based on age, language, sexual orientation, relationship status, education, and employment status. Here education and employment status were included since matching based on age alone did not eliminate differences in education and employment status. Demographics were compared between groups using X^2^-tests in case of nominal scales, Mann-Whitney-*U*-Tests or Kruskall-Wallis-tests in case of ordinal scales, as well as *t*-tests or one-way-ANOVAs in case of continuous scales and *p*-values were FDR-corrected for multiple comparisons.

In order to assess whether personality and/or gender role related to participant’s choice to use HC, personality measures and gender role measures were compared using one-way ANOVAs between current HC-users, previous HC-users and never-users of HC. Independent samples *t*-tests were used to compare potential future HC-users and women, who said future HC-use was not an option for them, as well as IUD users and their matched group of COC-users. An FDR-correction of *p*-values for multiple comparisons was employed across all scales. Differences between sub-factors were explored, when a significant difference in the main scale was observed. As measures of effect size, η^2^ or Cohen’s d was calculated for each scale. In case significant differences were observed, we additionally calculated Mahalanobis D, which is a multivariate measure of effect size (Del Giudice, 2017) and has been previously used to compare the difference in personality profiles between groups (Del Giudice et al., 2012). Like in these previous studies, Mahalanobis D was calculated based on the averaged covariance matrix of both groups using the *maha* function of the *GenAlgo* packages in R 4.0.5 (Mardia et al., 1979). Exploratory *t*-tests were performed to compare personality, gender role and demographics between previous HC-users with and without emotional side effect. The dataset is available upon request from the corresponding author.

## Results

### Demographics

Demographic information of current, previous and never-users of HC prior to and after matching can be found in Tables 1, 2, respectively. Prior to matching, previous HC-users were on average older than current users, current and previous users were more likely in a relationship and more never-users and previous users had children than current users. These data correspond to women’s contraceptive history, with contraceptive use during adolescence and young adulthood followed by a period of family planning. Furthermore, HC-use was more common among heterosexual than homosexual women. Average age was around 25 years, education level was generally high and increased with older age and employment status varied. Accordingly, the sample was representative of the university population from which participants were recruited. After matching, no differences between the current, previous and never-users of HC remained in demographic variables. Comparisons of prospective HC-users and their matched group of women, who do not intend to ever use HC, as well as current IUD-users and their matched group of current COC-users can be found in Supplementary Tables 1, 2, respectively. After matching, no differences in demographic variables were observed between the groups, with the exception of women for whom HC-use was a future option being in a relationship significantly more often than women who do not intend to ever use HC.

**TABLE 2:** Comparison of demographic variables between current and previous HC-users and never users matched for age, language, sexual orientation, and relationship status.

		Never-users (*n* = 321)	Previous users (*n* = 321)	Current users (*n* = 321)	Comparison
Language*^a^*	Lithuanian	97 (17%)	71 (22%)	94 (29%)	*p* = 0.248
	German	224 (83%)	250 (78%)	227 (71%)	
Age*^b^*		24.85 ± 5.82	24.98 ± 5.56	24.85 ± 6.90	*p* = 0.954
Handedness*^a^*	Left-handed	35 (11%)	28 (9%)	24 (7%)	*p* = 0.441
Education*^c^*	Apprenticeship	3 (1%)	4 (1%)	2 (1%)	*p* = 0.248
	Middle school	4 (1%)	5 (1%)	11 (3%)	
	High school	143 (46%)	136 (43%)	155 (48%)	
	University	158 (51%)	175 (55%)	143 (45%)	
	Unknown	13 (4%)	1 (0%)	2 (0%)	
Employment status*^c^*	Employed full time	67 (21%)	71 (22%)	90 (28%)	*p* = 0.248
	Employed part time	51 (16%)	46 (14%)	64 (20%)	
	In education + part time	31 (10%)	63 (20%)	22 (7%)	
	In education + unempl.	62 (19%)	40 (12%)	63 (20%)	
	Unemployed	110 (34%)	101 (32%)	104 (32%)	
Sexual orientation*^a^*	Homosexual	18 (6%)	13 (4%)	18 (6%)	*p* = 0.297
	Bisexual	72 (22%)	92 (29%)	109 (34%)	
	Heterosexual	231 (72%)	216 (67%)	194 (60%)	
Relationship	In a relationship*^a^*	179 (56%)	205 (64%)	188 (59%)	*p* = 0.248
	Duration*^b^*	5.06 ± 4.72	4.28 ± 4.66	5.28 ± 5.88	*p* = 0.248
	Satisfaction*^b^*	8.60 ± 1.65	8.52 ± 1.45	8.46 ± 1.55	*p* = 0.863
Children*^a^*		42 (13%)	37 (12%)	40 (12%)	*p* = 0.954

*^a^*binary variables, compared via *X*^2^-Tests, *^b^*continuous variable, compared via one-way-ANOVAs, *^c^*ranked variables, compared via Kruskall-Wallis tests. *p*-values were FDR-corrected for multiple comparisons.

### Health variables, hormonal contraceptive-use and side effects

Comparison of health variables and HC characteristics between current, previous and never-users are summarized in Table 3. Health information for prospective HC-users and IUD-users is included in Supplementary Table 2. Groups did not differ in health variables, with the exception of alcohol consumption being more common among current and previous HC-users compared to never users.

**TABLE 3:** Comparison of health variables and HC-characteristics between current and previous HC-users and never users matched for age, language, sexual orientation, and relationship status.

		Never-users (*n* = 321)	Previous users (*n* = 321)	Current users (*n* = 321)	Comparison
Health*^a^*	Smokers	28 (9%)	43 (14%)	29 (9%)	*p* = 0.325
	Alcohol	132 (41%)	179 (55%)	195 (61%)	***p*** ** < 0.001**
	Medication	35 (11%)	46 (14%)	50 (16%)	*p* = 0.325
	Neurological disorder	6 (2%)	7 (2%)	3 (1%)	*p* = 0.493
	Psychological disorder	28 (9%)	42 (13%)	24 (7%)	*p* = 0.325
	Endocrine disorder	23 (7%)	38 (12%)	20 (6%)	*p* = 0.092
	Heart disease	8 (3%)	5 (2%)	10 (3%)	*p* = 0.493
	Stress	142 (44%)	142 (44%)	155 (48%)	*p* = 0.493
Characteristics of HC-use	Number of different HC*^b^*	N/A	1.12 ± 0.34	1.36 ± 0.58	***p*** ** < 0.001**
	Duration of use*^b^*		3.36 ± 2.69	4.73 ± 4.45	***p*** ** < 0.001**
	Age at first use*^b^*		17.81 ± 3.03	19.45 ± 5.30	***p*** ** < 0.001**
	Time since discontin.		4.04 ± 4.31	N/A	
	Start dur. adolescence*^a^*		263 (82%)	247 (77%)	***p*** ** = 0.010**
	Start with relationship*^a^*		82 (25%)	135 (42%)	***p*** ** < 0.001**
Type of HC*^a^*	COC	N/A	306 (95%)	233 (73%)	***p*** ** < 0.001**
	IUD		1 (0%)	57 (18%)	
	Ring		7 (2%)	22 (7%)	
	Other		4 (1%)	9 (3%)	
Main reason for HC-use*^a^*	Contraception	N/A	223 (69%)	212 (66%)	***p*** ** < 0.001**
	Menstrual pain		25 (7%)	65 (20%)	
	Menstrual cycle control		22 (7%)	6 (2%)	
	Acne		19 (6%)	12 (4%)	
	PCOS		8 (2%)	11 (3%)	
	Endometriosis		2 (1%)	10 (3%)	
	PMS		2 (1%)	0 (0%)	
	Other		12 (4%)	5 (2%)	
Side effects*^a^*	Psychological	N/A	174 (54%)	9 (3%)	***p*** ** < 0.001**
	Weight changes		72 (22%)	2 (1%)	***p*** ** < 0.001**
	Other		62 (19%)	3 (1%)	***p*** ** < 0.001**
	Loss of libido		70 (22%)	74 (23%)	*P* = 0.958
	Bleeding		30 (9%)	39 (12%)	*P* = 0.398
	Headaches		33 (10%)	43 (13%)	*p* = 0.390
	Nausea		28 (9%)	24 (7%)	*p* = 0.477
	Breast pain		13 (4%)	25 (8%)	*P* = 0.084
	Skin changes		20 (6%)	42 (13%)	***p*** ** = 0.010**
Reason not to use HCs*^a^*	No need	53 (17%)	31 (9%)	N/A	***p*** ** < 0.001**
	Medical reasons	10 (3%)	19 (6%)		
	Negative experiences	N/A	186 (58%)		
	Worried about side/long-term effects	220 (69%)	69 (22%)		
	Opposed to hormones	36 (11%)	15 (4%)		
	Other	1 (0%)	3 (1%)		
Future HC-use optional*^a^*		41 (13%)	24 (8%)	N/A	*p* = 0.082

*^a^*Binary variables compared via *X*^2^-tests, *^b^*continuous variables compared via t-tests. *p*-values were FDR-corrected for multiple comparisons, separately for health variables, HC-characteristics and discontinuation characteristics. Bold *p*-values indicate significant difference.

Current users had used significantly more different HCs than previous users, had used HC for a longer period of time and had started their HC later, resulting in more adolescent starters (before the age of 21) than previous users. Use of COC was significantly more common among previous users compared to current users, while IUD-use was significantly more common among current users. However, when also considering the contraceptives previously used by current users, 91% of those not currently on COC had previously used COC.

Current users reported significantly more commonly that they had started HC-use when they entered a new relationship, though contraception was the most common reason for HC-use in both groups. Current users reported more HC-use for the treatment of gynecological problems like menstrual pain, polycystic ovary syndrome (PCOS) or endometriosis, while previous HC-users reported more HC-use for non-contraceptive benefits, like menstrual cycle control or the treatment of acne. Previous users also listed significantly more other reasons for their HC-use, including non-specified medical reasons, and advice to use HC by parents or gynecologists.

Side effects were significantly more common among previous users than current users. The most pronounced difference emerged for psychological side effects (mood swings, depressed mood, anxiety, and irritability), even though neither current users nor previous users were specifically asked for psychological side effects. While 3% of current users reported psychological side effects of their own accord, 54% of the previous users listed psychological side effects, most commonly also as reason not to use HCs. Furthermore, weight changes and other medical side effects (hair loss, swollen legs, vaginal dryness, edema, thrombosis, etc.) were significantly more common among previous users compared to current users. Loss of libido and physical side effects (headaches, nausea, and breast pain) were comparable between current and previous HC-users, while skin changes were more commonly reported in current OC-users. However, 2% of current users listed positive rather than negative skin changes.

Regarding the reasons not to use HC among current non-users, never-users more frequently reported concern about potential side effects or a general opposition to hormones, while previous users mostly listed negative experiences with HC as reason not to use HC again. The percentage of prospective future HC-users was comparable among never-users and previous users.

### Personality and choice of hormonal contraceptive

For the HEXACO-60, average scale scores were slightly above the scale mean of 3 (honesty/humility: 3.88 ± 0.43; emotionality: 3.59 ± 0.48, extraversion: 3.44 ± 0.48, agreeableness: 3.45 ± 0.47, conscientiousness: 3.86 ± 0.45, openness: 3.62 ± 0.42), which is in accordance with values reported for women in the original English version of the HEXACO-60 (Ashton and Lee, 2009), as well as the German and Lithuanian versions of the HEXACO-60 (Truskauskaitė-Kunevičienė et al., 2012; Moshagen et al., 2014). Accordingly, the sample is representative with respect to personality.

Significant differences between current, previous and never users of HC in personality and gender role emerged only with respect to the Honesty-Humility scale of the HEXACO-60 (see Table 4). Subscale-analyses revealed that this difference was driven by the Greed-Avoidance subscale. Sidak *post hoc* comparisons revealed that the difference emerged between current users and never-users (Honesty-Humility: *p* = 0.002; Greed Avoidance: *p* < 0.001), with previous users taking intermediate values with no significant differences to the other groups (all *p* < 0.150). Note, however, that the effect size for this difference was small (Honesty-Humility: η^2^ = 0.012, Greed Avoidance: η^2^ = 0.015). Mahalanobis D also amounted to very small effects sizes of 0.07 for comparison of previous to never-users and 0.15 for comparison of current to never-users.

**TABLE 4:** Personality differences between current, previous, and never-users of HC.

	Never users (*n* = 321)		Previous users (*n* = 321)		Current users (*n* = 321)		Comparison			
				
	Mean	SD	Mean	SD	Mean	SD	F	P	p_FDR_	η^2^
**Honesty-humility**	**3.93**	**0.43**	**3.87**	**0.42**	**3.82**	**0.41**	**5.94**	**0.003**	**0.021**	**0.012**
***Greed avoidance***	**3.67**	**0.69**	**3.57**	**0.66**	**3.47**	**0.63**	**7.52**	**0.001**	**0.014**	**0.015**
*Fairness*	4.11	0.62	4.06	0.61	3.98	0.62	3.98	0.019	0.077	0.008
*Sincerity*	3.69	0.79	3.64	0.81	3.62	0.78	0.75	0.472	0.601	0.002
*Modesty*	4.28	0.57	4.26	0.62	4.22	0.57	0.73	0.481	0.601	0.002
Emotionality	3.54	0.48	3.57	0.49	3.59	0.45	0.85	0.426	0.601	0.002
Extraversion	3.38	0.48	3.45	0.47	3.42	0.51	1.64	0.194	0.452	0.003
Agreeableness	3.51	0.47	3.46	0.46	3.41	0.47	3.84	0.022	0.077	0.008
Conscientiousness	3.83	0.47	3.86	0.42	3.88	0.46	0.98	0.377	0.601	0.002
Openness	3.63	0.42	3.65	0.41	3.62	0.43	0.58	0.558	0.601	0.001
GERAS_femininity*^a^*	4.91	0.58	4.93	0.61	4.90	0.59	0.21	0.808	0.808	<0.001
GERAS_masculinity*^a^*	3.97	0.64	3.98	0.66	4.03	0.70	0.76	0.470	0.601	0.002
SIS_femininity*^a^*	6.39	1.38	6.52	1.50	6.43	1.50	0.60	0.548	0.601	0.001
SIS_masculinity*^a^*	2.57	1.56	2.55	1.69	2.81	1.79	2.24	0.107	0.300	0.005

^*a*^Please not that gender role ratings were missing from 6 current users, 13 previous users and 8 never-users. GERAS, Gender-related attributes questionnaire; SIS, Six-Item-Scale; pFDR, FDR-corrected *p*-value; η^2^, estimate of effect size.

Furthermore, no significant differences in personality or gender role were observed between non-users, who saw HC-use as a viable option for the future and non-users, who were strictly opposed to HC-use (compare Table 5). We did notice, however, that due to the smaller sample size, effect sizes for this comparison were in part larger than effect sizes for the comparisons of current HC-users and non-users, which yielded significant results. Among the personality dimensions, effect sizes were larger than 0.20 for Honesty-Humility and conscientiousness. Honesty-Humility was higher in women, for whom future HC-use was not an option, while conscientiousness was higher in women, for whom future HC-use was an option. Mahalanobis D for this comparison was 0.24, which also corresponds to a small effect size.

**TABLE 5:** Personality differences between non-users who did and did not see the use of HC as a potential future option matched for age, language, sexual orientation, and relationship status.

	HC no opt. (*n* = 73)		HC option (*n* = 73)		Comparison		
			
	Mean	SD	Mean	SD	*t*	*p*	*d*
**Honesty-humility**	**3.99**	**0.39**	**3.87**	**0.43**	**1.75**	**0.082**	**0.29**
Emotionality	3.52	0.50	3.59	0.51	–0.74	0.463	0.12
Extraversion	3.39	0.40	3.36	0.46	0.42	0.673	0.07
Agreeableness	3.51	0.49	3.55	0.50	–0.45	0.650	0.08
**Conscientiousness**	**3.81**	**0.46**	**3.94**	**0.49**	**−1.61**	**0.109**	**0.27**
Openness	3.61	0.40	3.59	0.39	0.23	0.819	0.04
GERAS_femininity*^a^*	4.88	0.55	4.82	0.57	0.62	0.538	0.11
GERAS_masculinity*^a^*	4.05	0.62	3.89	0.63	1.46	0.145	0.26
SIS_femininity*^a^*	6.70	1.40	6.36	1.62	1.31	0.192	0.22
SIS_masculinity*^a^*	2.30	1.70	2.51	1.65	–0.77	0.444	0.13

^*a*^Please note that gender role ratings were missing from 2 women in each group. GERAS, Gender-related attributes questionnaire; SIS, Six-Item-Scale; d, Cohen’s d.

Finally, the comparison of current IUD-users and a matched sample of COC-users demonstrated significantly higher extraversion (sociability) and significantly higher agreeableness (forgiveness) among IUD-users compared to COC-users. Effect sizes for these comparisons were moderate with Cohen’s d ranging from 0.38 to 0.49. Likewise, Mahalanobis D across all HEXACO-60 dimensions was 0.45 for this comparison (compare Table 6).

**TABLE 6:** Personality differences between IUD-users and COC-users matched for age, language, sexual orientation, relationship status, education, and employment status.

	COC (*n* = 94)		IUD (*n* = 94)		Comparison			
			
	Mean	SD	Mean	SD	*t*	*p*	p_FDR_	*d*
Honesty-humility	3.83	0.37	3.89	0.44	−0.91	0.344	0.476	−0.15
Emotionality	3.51	0.43	3.57	0.51	−0.84	0.404	0.519	−0.13
**Extraversion**	**3.34**	**0.45**	**3.57**	**0.60**	−**2.99**	**0.003**	**0.018**	−**0.43**
*Self esteem*	3.80	0.50	3.90	0.61	−1.22	0.223	0.365	−0.18
*Social boldness*	3.21	0.72	3.38	0.79	−1.58	0.116	0.236	−0.22
***Sociability***	**2.61**	**0.95**	**3.11**	**1.10**	−**3.38**	**0.001**	**0.018**	−**0.49**
*Liveliness*	3.56	0.60	3.80	0.73	−2.46	0.015	0.054	−0.36
**Agreeableness**	**3.30**	**0.48**	**3.50**	**0.42**	−**2.98**	**0.003**	**0.018**	−**0.44**
***Forgiveness***	**2.54**	**0.98**	**2.91**	**0.98**	−**2.59**	**0.010**	**0.045**	−**0.38**
*Patience*	3.65	0.80	3.88	0.67	−2.12	0.035	0.105	−0.31
*Gentleness*	3.30	0.66	3.45	0.62	−1.59	0.114	0.236	−0.23
*Flexibility*	3.57	0.50	3.68	0.47	−1.46	0.147	0.265	−0.23
Conscientiousness	3.89	0.46	3.84	0.45	0.77	0.441	0.529	0.11
Openness	3.58	0.40	3.65	0.41	−1.11	0.268	0.402	−0.17
GERAS_femininity^a^	4.85	0.63	4.89	0.60	−0.37	0.713	0.802	−0.07
GERAS_masculinity^a^	3.91	0.68	4.06	0.66	−1.57	0.118	0.236	−0.22
SIS_femininity^a^	6.50	1.33	6.53	1.63	−0.16	0.875	0.926	−0.02
SIS_masculinity^a^	2.84	1.79	2.82	1.80	0.06	0.951	0.951	0.01

^a^Please note that gender role ratings were missing from 1 woman in each group. GERAS, Gender-related attributes questionnaire; SIS, Six-Item-Scale; d, Cohen’s d; COC, combined oral contraceptives; IUD, intra-uterine device.

### Side effects and personality

An exploratory comparison of previous HC-users with and without emotional side effects, revealed no significant differences in personality or gender role with no effect sizes larger than 0.20 (all *t* < 1.80, all *p* > 0.07). However, we did observe a number of interesting demographic differences between women with previous emotional symptoms and women without previous emotional symptoms (compare Table 7). Previous adverse emotional side effects were significantly more often reported by women from Germany and Austria than women from Lithuania. Also, women who reported previous adverse emotional side effects were significantly younger and accordingly had lower education, shorter relationship durations and fewer children. Interestingly, the two groups did not differ in any of the health variables, including psychological disorders and stress and no differences were observed in the reasons for HC-use.

**TABLE 7:** Comparison of previous HC-users with and without emotional symptoms along demographic variables.

		No previous mood symptoms (*n* = 217)	Previous mood symptoms (*n* = 254)	Comparison
Language	Lithuanian	61 (28%)	26 (10%)	***p*** ** < 0.001**
	German	156 (72%)	228 (90%)	
Age		29.31 ± 7.09	25.64 ± 5.41	***p*** ** < 0.001**
Handedness	Left-handed	13 (6%)	27 (11%)	*p* = 0.174
Education	Apprenticeship	4 (2%)	5 (2%)	*p* = 0.017
	Middle school	2 (1%)	2 (1%)	
	High school	55 (26%)	97 (38%)	
	University	154 (71%)	150 (59%)	
	Unknown	1 (0%)	0 (0%)	
Employment status	Employed full time	77 (36%)	58 (23%)	*p* = 0.077
	Employed part time	47 (22%)	57 (22%)	
	In education + Minor employment	22 (10%)	46 (18%)	
	In education + no employment	19 (9%)	34 (13%)	
	Unemployed	52 (24%)	59 (23%)	
Sexual orientation	Homosexual	10 (5%)	4 (2%)	*p* = 0.700
	Bisexual	44 (20%)	70 (28%)	
	Heterosexual	163 (75%)	180 (71%)	
Relationship	In a relationship	162 (75%)	188 (74%)	*p* = 1.000
	Duration	7.13 ± 5.93	4.45 ± 4.88	***p*** ** < 0.001**
	Satisfaction	8.58 ± 1.56	8.52 ± 1.50	*p* = 0.880
Children		58 (27%)	26 (10%)	*p* < 0.001
Health	Smokers	30 (14%)	25 (10%)	*p* = 0.303
	Alcohol	122 (56%)	135 (53%)	*p* = 0.700
	Medication	45 (21%)	36 (14%)	*p* = 0.149
	Neurological disorder	10 (5%)	4 (2%)	*p* = 0.149
	Psychological disorder	26 (12%)	32 (13%)	*p* = 1.000
	Endocrine disorder	36 (17%)	30 (12%)	*p* = 0.242
	Heart disease	4 (2%)	5 (2%)	*p* = 1.000
	Stress	101 (47%)	119 (47%)	*p* = 1.000
Previous HC-use	Duration of use	4.72 ± 3.88	4.27 ± 3.45	*p* = 0.185
	Age at first use	18.47 ± 4.09	17.84 ± 3.21	*p* = 0.164
	Adolescent start (<21)	159 (78%)	211 (85%)	*p* = 0.149
	Time since discontin.	6.05 ± 5.81	4.01 ± 3.65	***p*** ** < 0.001**
Reason for Previous HC-Use	Contraception	145 (67%)	194 (76%)	*p* = 0.164
	Menstrual cycle control	19 (9%)	9 (4%)	
	Menstrual pain	15 (7%)	18 (7%)	
	Acne	11 (5%)	16 (6%)	
	Other	17 (8%)	12 (5%)	
Reasons not to use HCs	No need	39 (18%)	6 (2%)	***p*** ** < 0.001**
	Medical reasons	17 (8%)	12 (5%)	
	Negative experiences	76 (35%)	202 (80%)	
	Worry about side-effects	61 (28%)	27 (11%)	
	Opposed to hormones	22 (10%)	4 (2%)	
	Other	2 (1%)	3 (1%)	

*P*-values were FDR-corrected for multiple comparisons. Bold *p*-values indicate significant difference.

## Discussion

The aim of the present study, was to identify personality factors associated with women’s choice to use or not use HC and the type of HC chosen. The results demonstrate only a very weak association between the willingness to use or not use HC and the *Greed Avoidance* subscale of the *Honesty-Humility* scale, while no association between the classical BIG-5 personality factors (emotionality/neuroticism, extraversion, agreeableness, conscientiousness, openness) or gender role and HC-use was observed. However, participant’s personality profile was significantly associated with the type of HC chosen. We observed higher agreeableness and extraversion in users of IUD compared to users of COC. In the following we will first discuss the personality characteristics associated with HC-use and HC-type in more detail and then discuss our exploratory findings regarding adverse emotional side effects.

The fact that participants willingness to use HC was not associated with the BIG-5 or gender role, is in contrast to previous studies suggesting association between HC-use and neuroticism or extraversion (Beard et al., 1974; Priestnall et al., 1978; Jacobsson et al., 1981; Ross et al., 2001), as well as femininity (Pletzer et al., 2015). However, these studies did not differentiate between never users, previous users, and prospective users in the group of naturally cycling women, had smaller sample sizes and did not match HC-users and naturally cycling women for demographic variables or relationship status. Furthermore, the most recent studies reported no associations between HC-use and personality on the one hand (Beltz et al., 2019) or gender role on the other hand (Nielson and Beltz, 2021). These results suggest that if demographic variables and relationship status are controlled for, personality and gender role do not present additional confounds for neurocognitive research on HC. The exception is a small association between the willingness to use HC and lower scores on the *Greed Avoidance* subscale of the *Honesty-Humility* scale. According to the HEXACO authors, *Greed Avoidance* assesses a tendency to be uninterested in signs of high social status (Lee and Ashton, 2009). In the items associated with this scale, current and prospective HC-users reported a higher interest in money and luxury goods compared to non-users. Though only a speculation, one explanation for this finding could be the socio-economic consequences of an unplanned pregnancy (Lersch et al., 2017).

Regarding HC type, IUD-users score higher on the *Forgiveness* subscale of the *Agreeableness* scale and the *Sociability* subscale of the *Extraversion* scale. The findings on both scales may be related as they both hint at a more positive attitude toward social interactions in IUD-users compared to COC-users. According to the HEXACO authors, people with high sociability scores enjoy talking, visiting, and celebrating with others. A more permanent HC option may facilitate the participation in a variety of social activities without having to remember the daily intake regimen all the time. In light of this interpretation, it is an interesting observation, that the other personality factor that we hypothesized to differ, i.e., conscientiousness, does not appear to contribute to the choice of HC type.

It is noteworthy, that the difference in *Greed Avoidance* emerged only between current users of HC and never users of HC, not between previous users and never-users. A trend in the same direction was also observed for prospective users of HC. This hints at a slightly stronger association between personality characteristics and the willingness to use HC now as compared to about 10 years ago, when previous users had on average started HC (compare Table 3). This result fits with the observation of a shift in attitudes toward HC (Fiala and Parzer, 2019; Svahn et al., 2021). While 10 years ago, HC was the standard contraceptive choice in Europe and the US, women consider their contraceptive options more carefully today. This interpretation is in line with the results, that current users take their HC primarily for contraceptive and gynecological reasons, while previous users also list a number of non-contraceptive benefits, like menstrual cycle regulation or the treatment of acne. Furthermore, previous users frequently name parents and gynecologists to have recommended HC-use among other reasons to use HC, while current non-users frequently list friends who recommended not to use HC among other reasons not to use HC. The reasons listed not to use HC included negative experiences, worry about side effects, medical reasons or an opposition to synthetic hormones. This compares to a recent systematic review on the reasons for rejecting HC in western countries (Le Guen et al., 2021). Interestingly, while adverse emotional side effects are frequently named by current and previous users of HC, the treatment of premenstrual syndrome (PMS) is rarely mentioned as a reason for HC-use.

We did indeed observe some interesting results regarding emotional side effects of HC, although information on adverse mood effects was not specifically requested from the participants, but entered of their own accord in an open answer field. Adverse emotional side effects were reported significantly more often by previous users than current users. While the frequency of emotional side effects in current users, i.e., 3%, is a little lower than the rate observed in prospective randomized controlled trials (Lundin et al., 2017), more than half of the previous users report mood swings, depressed mood, increased emotionality, irritability, and/or anxiety. This observation is in line with various studies demonstrating reduced positive affect and altered stress responsivity in HC-users (Sanders et al., 2001; Nielsen et al., 2013, 2014; Lewis et al., 2019; Gervasio et al., 2022) and that these side effects have been associated with discontinuing usage (Lindh et al., 2009; Sundström-Poromaa and Segebladh, 2012; Sundström-Poromaa, 2021). Indeed, adverse side effects were often cited as reason not to use HC and not to consider HC-use in the future in the current sample. Accordingly, our observation of higher adverse mood symptoms among previous users compared to current users may be reflective of the well-known “survivor-effect” (Oinonen and Mazmanian, 2002). Due to the retrospective nature of this study, the mechanisms underlying these associations between HC-use and emotional side effects remain to be elucidated. Please note also, that this questionnaire was administered during the Covid-19 pandemic to a sample consisting of mostly university students. Therefore, though data were collected during a time when no COVID-lockdowns were in place in any of the participating countries, the added stress of the pandemic, distance learning and online examinations may have contributed to the reporting of adverse mood effects.

Finally, the socio-demographic differences between women, who did and did not report previous adverse mood effects suggest a shift in how often adverse mood symptoms are attributed to HC. For example, previous HC-users in Lithuania report less adverse mood effects than those in Austria and Germany. Furthermore, younger women, who discontinued their HC more recently (compare Table 7) report more adverse mood effects. Apparently, the experience/reporting of adverse mood effects is susceptible to cultural and generational context. It appears that the number of negative mood symptoms attributed to HC has increased. Whether this observation is reflective of increased education about side effects and different contraceptive options resulting in different perceptions of HC across generations or a shift in the prescribed HC formulations with different side effect profiles cannot be determined based on these retrospective reports. Importantly, age at first HC-use did not differ significantly between previous users with and without emotional side effects. This is in contrast to previous observational studies suggesting stronger emotional side effects in adolescent starters (e.g., Skovlund et al., 2016).

There are several methodological aspects that are important to consider when interpreting the results of present study. First, the current study relied on a comparably small sample size remaining for the assessment of future attitudes toward HC and HC type. A more convincing connection between personality and women’s attitudes toward HC could have been obtained from reliable results about future contraceptive choices. It is remarkable though, that the vast majority of women, who don’t use HC at the moment (∼90%), do not consider future HC-use as an option. It appears that the majority of women, who have neutral or positive attitudes toward HC, are already using them. Negative attitudes toward HC, however, primarily stem from concerns about their adverse effects, either due to personal negative experiences or from reports of others (friends, media, etc.). The large number of current HC-users, previous users and never-users and the close matching for demographic differences among these groups is, however, a major strength of the current study. Second, the sample of our study represents mostly the university population living in Austria, Germany, and Lithuania. Therefore, the absence of full demographic data regarding age, race/ethnicity, and socioeconomic status may limit generalizability. Third, despite the broad range of associations evaluated in the present study, we are not able to conclude whether the reported adverse mood effects were causally related to HC-use. A longitudinal study is needed for such evaluation. Finally, we were unable to control the environment and time spent to fill in questionnaires due to the setup of the study as an online survey.

In summary, associations between personality and the choice to use or not use HC were negligible, though the type of HC chosen was associated with personality traits. Accordingly, we do not expect confounding effects of personality on neurocognitive experiments regarding COC, provided that other demographic differences between COC-users and non-users are well controlled for. Cross-sectional studies comparing IUD-users and COC-users may, however, consider to take personality into account.

## Data availability statement

The raw data supporting the conclusions of this article are publicly available at https://osf.io/rmauq/.

## Ethics statement

The studies involving human participants were reviewed and approved by the Ethics Committee of the University of Salzburg. The patients/participants provided their written informed consent to participate in this study.

## Author contributions

BP, BD, and RG designed the study. CL created the online questionnaire. BP and RG organized the Lithuanian translation of the questionnaire. BP, BD, RG, and CL acquired the data. BP analyzed the data and wrote the first draft of the manuscript, which was revised and approved by BD and RG. All authors contributed to the article and approved the submitted version.
