# Supplementary material for: Weak associations between personality and contraceptive choice
Source: Front Neurosci. 2022 Oct 27;16:898487. doi: 10.3389/fnins.2022.898487 (PMC9648366; doi:10.3389/fnins.2022.898487)
Supplement: Supplementary file 1 [file Data_Sheet_1.docx]

**Supplementary Table 1**: Comparison of demographic and health variables between non-users for who future HC-use is and is not an option matched for age, language, sexual orientation, and relationship status. p-values were FDR-corrected for multiple comparisons.

|  |  | **HC-use no option**  **matched (n = 73)** | **HC-use option**  **(n = 73)** | **Comparison** |
| --- | --- | --- | --- | --- |
| Language | Lithuanian | 31 (42%) | 28 (38%) | p = 0.941 |
|  | German | 42 (58%) | 45 (62%) |  |
| Age |  | 25.62 ± 6.41 | 26.00 ± 6.30 | p = 0.941 |
| Handedness | Left-handed | 6 ( 8%) | 11 (15%) | p = 0.717 |
| Education | Apprenticeship | 0 ( 0%) | 1 ( 1%) | p = 0.424 |
|  | Middle school | 0 ( 0%) | 0 ( 0%) |  |
|  | High school | 20 (27%) | 26 (37%) |  |
|  | University | 52 (71%) | 44 (62%) |  |
|  | Unknown | 1 ( 1%) | 0 ( 0%) |  |
| Employment status | Employed full time | 24 (33%) | 22 (30%) | p = 0.424 |
|  | Employed part time | 13 (18%) | 6 ( 8%) |  |
|  | In education + part time | 11 (15%) | 7 (10%) |  |
|  | In education + unemploy. | 7 (10%) | 11 (15%) |  |
|  | Unemployed | 18 (25%) | 27 (37%) |  |
| Sexual Orientation | Homosexual | 2 ( 3%) | 5 ( 7%) | p = 0.442 |
|  | Bisexual | 11 (15%) | 16 (22%) |  |
|  | Heterosexual | 60 (82%) | 52 (71%) |  |
| Relationship | **In a relationship** | **11 (15%)** | **32 (43%)** | **p < 0.001** |
|  | Duration | 5.27 ± 3.52 | 6.47 ± 4.26 | p = 0.857 |
|  | Satisfaction | 8.73 ± 1.10 | 8.41 ± 1.64 | p = 0.872 |
| Children |  | 7 (10%) | 9 (12%) | p = 0.941 |
| Health | Smokers | 7 (10%) | 11 (15%) | p = 0.857 |
|  | Alcohol | 35 (48%) | 30 (42%) | p = 0.872 |
|  | Medication | 12 (16%) | 11 (15%) | p = 1.000 |
|  | Neurological disorder | 6 ( 8%) | 0 ( 0%) | p = 0.133 |
|  | Psychological disorder | 5 ( 7%) | 7 (10%) | p = 0.941 |
|  | Endocrine disorder | 3 ( 4%) | 13 (18%) | p = 0.095 |
|  | Heart disease | 1 ( 1%) | 1 ( 1%) | p = 1.000 |
|  | Stress | 33 (45%) | 31 (42%) | p = 1.000 |
| Reason not to use HCs | No need | 5 ( 7%) | ´73 (100%) | **p < 0.001** |
|  | Medical reasons | 8 (11%) | 0 ( 0%) |  |
|  | Worried about side effects | 54 (74%) | 0 ( 0%) |  |
|  | Opposed to hormones | 6 ( 8%) | 0 ( 0%) |  |
|  | Other | 2 ( 1%) | 0 ( 0%) |  |

**Supplementary Table 2**: Comparison of demographic and health variables between current IUD-users and current COC-users matched for age, language, sexual orientation, relationship status, education and employment status. p-values were FDR-corrected for multiple comparisons. COC = combined oral contraceptives, IUD = intra-uterine device

|  |  | **COC (n = 94)** | **IUD (n = 94)** | **Comparison** |
| --- | --- | --- | --- | --- |
| Language | Lithuanian | 32 (34%) | 29 (31%) | p = 0.720 |
|  | German | 62 (66%) | 65 (69%) |  |
| Age |  | 29.99 ± 5.71 | 28.43 ± 7.92 | p = 0.375 |
| Handedness | Left-handed | 6 ( 6%) | 10 (11%) | p = 0.553 |
| Education | Apprenticeship | 0 ( 0%) | 0 ( 0%) | p = 0.324 |
|  | Middle school | 0 ( 0%) | 4 ( 4%) |  |
|  | High school | 22 (23%) | 30 (32%) |  |
|  | University | 72 (77%) | 59 (63%) |  |
|  | Unknown | 0 ( 0%) | 0 ( 0%) |  |
| Employment status | Employed full time | 45 (48%) | 30 (32%) | p = 0.375 |
|  | Employed part time | 13 (14%) | 22 (23%) |  |
|  | In education + part time | 6 ( 6%) | 13 (14%) |  |
|  | In education + unemploy. | 13 (14%) | 6 (15%) |  |
|  | Unemployed | 17 (18%) | 23 (24%) |  |
| Sexual Orientation | Homosexual | 2 ( 3%) | 5 ( 7%) | p = 0.392 |
|  | Bisexual | 11 (15%) | 16 (22%) |  |
|  | Heterosexual | 60 (82%) | 52 (71%) |  |
| Relationship | In a relationship | 80 (85%) | 79 (85%) | p = 0.889 |
|  | Duration | 6.49 ± 5.82 | 7.15 ± 6.42 | p = 0.685 |
|  | Satisfaction | 8.36 ± 1.84 | 8.54 ± 1.76 | p = 0.688 |
| Children |  | 14 (14%) | 26 (28%) | p = 0.324 |
| Health | Smokers | 9 (10%) | 13 (14%) | p = 0.596 |
|  | Alcohol | 49 (52%) | 54 (57%) | p = 0.685 |
|  | Medication | 11 (12%) | 20 (21%) | p = 0.375 |
|  | Neurological disorder | 0 ( 0%) | 0 ( 0%) | p = 1.000 |
|  | Psychological disorder | 13 (14%) | 6 ( 6%) | p = 0.375 |
|  | Endocrine disorder | 6 ( 6%) | 8 ( 9%) | p = 0.694 |
|  | Heart disease | 4 ( 4%) | 1 ( 1%) | p = 0.392 |
|  | Stress | 51 (54%) | 44 (47%) | p = 0.553 |

**Supplementary Table 3:** ANCOVA results (F-values) comparing personality and gender role between the unmatched groups of current, previous and never-users of HC, while controlling for country, age, sexual orientation and relationship as covariates. H = Honesty-Humility, E = Emotionality, X = Extraversion, A = Agreeableness, C = Conscientiousness, O = Openness, GERAS_M = Masculinity Scale of the Gender-related attributes questionnaire, GERAS_F = Femininity scale of the gender-related attributes questionnaire, SIS_M = Masculinity scale of the six-item-scale, SIS_F = Femininity scale of the six-item-scale. *p ˂ 0.05, **p ˂ 0.01, ***p ˂ 0.001, p-values were FDR-corrected for multiple comparisons.

|  | **H** | **E** | **X** | **A** | **C** | **O** | **GERAS_M** | **GERAS_F** | **SIS_M** | **SIS_F** |
| --- | --- | --- | --- | --- | --- | --- | --- | --- | --- | --- |
| HC group | 4.83^*^ | 2.73 | 1.16 | 3.58 | 1.63 | 5.28^*^ | 0.09 | 1.28 | 1.37 | 0.10 |
| Country | 1.00 | 3.27 | 25.05^***^ | 0.28 | 0.30 | 0.59 | 19.57^***^ | 0.12 | 2.59 | 1.36 |
| Age | 1.46 | 39.41^***^ | 6.05^*^ | 3.24 | 0.28 | 6.48^*^ | 0.54 | 2.88 | 1.52 | 13.48^***^ |
| Sexual Orientation | 1.05 | 6.32^*^ | 4.13^*^ | 0.00 | 11.19^**^ | 12.52^***^ | 1.28 | 37.50^***^ | 105.99^***^ | 112.34^***^ |
| Relationship Status | 0.17 | 25.63^***^ | 0.23 | 0.70 | 0.00 | 0.58 | 0.05 | 7.42^**^ | 0.05 | 1.15 |
